# Peripapillary atrophy classification using CNN deep learning for glaucoma screening

**DOI:** 10.1371/journal.pone.0275446

**Published:** 2022-10-06

**Authors:** Abdullah Almansour, Mohammed Alawad, Abdulrhman Aljouie, Hessa Almatar, Waseem Qureshi, Balsam Alabdulkader, Norah Alkanhal, Wadood Abdul, Mansour Almufarrej, Shiji Gangadharan, Tariq Aldebasi, Barrak Alsomaie, Ahmed Almazroa

**Affiliations:** 1 Department of Imaging Research, King Abdullah International Medical Research Center, Riyadh, Saudi Arabia; 2 King Saud Bin Abdulaziz University for Health Sciences, Riyadh, Saudi Arabia; 3 Department of Biostatistics and Bioinformatics, King Abdullah International Medical Research Center, Riyadh, Saudi Arabia; 4 National Center for Artificial Intelligence (NCAI), Saudi Data and Artificial Intelligence Authority (SDAIA), Riyadh, Saudi Arabia; 5 Department of Optometry and Vision Sciences, College of Applied Medical Sciences, King Saud University, Riyadh, Saudi Arabia; 6 Department of Computer Engineering, College of Computer and Information Sciences, King Saud University, Riyadh, Saudi Arabia; 7 Department of Ophthalmology, King Abdulaziz Medical City, Ministry of National Guard-Health Affairs, Riyadh, Saudi Arabia; University Tunku Abdul Rahman, MALAYSIA

## Abstract

Glaucoma is the second leading cause of blindness worldwide, and peripapillary atrophy (PPA) is a morphological symptom associated with it. Therefore, it is necessary to clinically detect PPA for glaucoma diagnosis. This study was aimed at developing a detection method for PPA using fundus images with deep learning algorithms to be used by ophthalmologists or optometrists for screening purposes. The model was developed based on localization for the region of interest (ROI) using a mask region-based convolutional neural networks R-CNN and a classification network for the presence of PPA using CNN deep learning algorithms. A total of 2,472 images, obtained from five public sources and one Saudi-based resource (King Abdullah International Medical Research Center in Riyadh, Saudi Arabia), were used to train and test the model. First the images from public sources were analyzed, followed by those from local sources, and finally, images from both sources were analyzed together. In testing the classification model, the area under the curve’s (AUC) scores of 0.83, 0.89, and 0.87 were obtained for the local, public, and combined sets, respectively. The developed model will assist in diagnosing glaucoma in screening programs; however, more research is needed on segmenting the PPA boundaries for more detailed PPA detection, which can be combined with optic disc and cup boundaries to calculate the cup-to-disc ratio.

## Introduction

Globally, at least 2.2 billion people have vision impairment, with 1 billion cases classified as moderate-to-severe. Glaucoma is the leading cause of irreversible vision impairment worldwide [1]. However, these estimates remain uncertain, because they are associated with a two-fold confidence interval difference [2]. Tham et al. [3] estimated that 111.8 million glaucoma cases will be diagnosed by 2040 (79.76 million with primary open-angle glaucoma and 32.04 million with primary angle-closure glaucoma). In the United States, the prevalence of glaucoma is expected to increase to 4.2 million by 2030. Researchers have estimated that 50% of people in the US remain undiagnosed. Thus, if current screening methods are continued, 2.1 million US citizens will remain undiagnosed by 2030 [4]. Telemedicine innovations can overcome glaucoma screening shortcomings and may facilitate the detection of early-stage cases.

Peripapillary atrophy (PPA) is a morphological symptom of glaucoma, and its presence may be used to make an early diagnosis of glaucoma. PPA is one of the early clinical signs associated with glaucoma; an increase in PPA size is associated with disease progression [5–7]. Thus, automatic detection of PPA will be a valuable technique for diagnosing glaucoma in early stages in a timely and cost-effective manner, particularly in primary clinics or remote places where experts and equipment are often lacking. This study was aimed at developing a deep learning system that functions as a screening and diagnostic tool that can help practitioners detect PPA from fundus images.

This paper is organized as follows: the proposed methodology, dataset details, preprocessing technique, and the proposed convolutional neural network (CNN) model specifications are presented in Section 2. In Section 3, the protocol for evaluating the implementation details and evaluation metrics is discussed. The results of the work are presented in Section 4 and discussed in detail in Section 5. Finally, the study is summarized in Section 6 (Conclusions).

### Background

PPA is characterized by morphological changes in the chorioretinal areas surrounding the optic disc. Two types of PPA can be present: the alpha and beta zones [8]. The beta zone is seen as a crescent shape, adjacent to the optic disc (OD) temporally. It is characterized by atrophy of the pigment epithelium and choriocapillaris, resulting in a visible sclera. The alpha zone is characterized by irregular pigmentation (hyperpigmentation or hypopigmentation) of the retinal pigment epithelium (RPE) and thinning of the chorioretinal layer, located further away from the disc, adjacent to the retina on its periphery and to the beta zone on the central side [8].

PPA was significantly more prevalent in eyes with glaucoma than in normal eyes and in the beta zone than in the alpha zone. In early glaucomatous eyes, the alpha zone occupied 23.7% of the area, while the beta zone occupied 41.7%. In advanced-stage glaucoma, these numbers increased to 44.4% and 85.7%, respectively [8]. Jonas [5] indicated that observing PPA while assessing the condition of glaucoma can be useful for distinguishing open-angle glaucoma types. Additionally, it can be used to differentiate between normal and open-angle glaucoma because research has shown that the beta zone is usually the largest in those with normal-angle glaucoma [5]. PPA is a likely indicator of current, past, or future risk of OD splinter hemorrhage incidence [9].

PPA can be diagnosed using various imaging techniques, including color fundus photography, where a camera attached to a lens system takes a high-quality photograph of the retina, from which information about the location, size, and color of PPA can be obtained. Optical coherence tomography (OCT) is another imaging technique that provides cross-sectional images, which provide information on the size, location, and thickness of the retinal and subretinal layers. However, PPA detection is largely performed using fundus tomography rather than OCT owing to its ease of use in identifying the PPA region. Color fundus photographs provide a clearer view of textures due to its color intensity, therefore PPA manifests clearer. While, cross-sectional images obtained by OCT might introduce difficulty to the problem at hand, such as PPA detection.

### Related work

Many studies on segmenting the OD and optic cup boundaries have been reported, in which fundus images have been used to calculate the cup-to-disc ratio and traditional image processing techniques and deep learning approaches [10–16] or machine learning approaches [17–20] have been used to perform glaucoma classification. However, research on PPA is limited. Cheng et al. [21] designed a model using biologically inspired features (BIFs), which mimics how the cortex operates for visual perception by including scene classification and gait recognition approaches for the region where PPA acts. The designed model segments the focal region from retinal fundus images using threshold-based segmentation and detects the presence of PPA using a support vector machine (SVM). The accuracy was ~90%. Moreover, this study included a sample of myopic children only, and the detection of negative images (no PPA) was better than that of positive images (PPA). This is because negative images are fairly similar because they are from healthy individuals, whereas positive images depend on the progression of PPA. Septiarini et al. [22] proposed a model in which the ROI was located and segmented using image processing techniques (thresholding). The model also included a preprocessing phase, followed by feature extraction. Classification was performed using a backpropagation neural network (BPNN). This model achieved an overall accuracy of 96%, whereas in severe cases of PPA, the accuracy reached 100%. Sharma et al. [23] used a combination of statistical features and ResNet50 to produce a system with an accuracy of 95.83%. An advantage of their study was that the dataset was relatively large and diverse. Muramatsu et al. [24] used texture analysis to identify PPA after identifying the ROI using a p-tile thresholding method. The sensitivity was 73%, because there were only 26 PPA photos, all in moderate-to-severe stages. A limitation was that the ROI did not always include the full PPA area.

## Methodology

### Overview

The study was reviewed and approved by the ethics committee of King Abdullah International Medical Research Center (IRB number RC20/087/R). However, the committee waived consent because the data were images with anonymized personal data.

The proposed deep learning algorithm consists of two stages: localization of the optic nerve head (ONH) and classification of the stated region. The developed algorithm automatically functions for both tasks without the need for handcrafted techniques, Hence, it provides a fully automatic PPA screening approach. Additionally, while implementing the proposed algorithm, datasets from various distributions were used, which provides the ability to obtain a general screening software developed with images of different quality and variance PPA states. The main contributions of this study are as follows.

- Using fundus images from different public sources and a challenging local dataset to provide a the variety of images quality.
- The images were labeled as either PPA or non-PPA.
- Developing a screening methodology for localizing the region of interest within fundus images and performing a classification process for detecting PPA with an end-to-end deep learning algorithm without using any hand-crafted technique.
- Evaluating the classification algorithm with different performance measures, such as accuracy, balanced accuracy, precision, recall, F1-score, and performing ROC analysis.
- Conducting cross-validation evaluation for reliability assessment of the classification algorithm.

**Fig 1:**
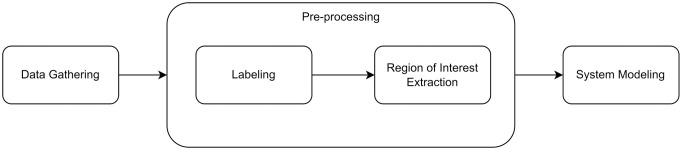
The process flow of the proposed PPA detection system.

The proposed detection system for the presence of PPA symptoms in fundus images classifies incoming fundus images into two groups: images stated as PPA and those that were not. Fig 1 outlines the process of designing such systems. The initial step involves data gathering, followed by a preparation stage in accordance with the system design. A classification process was then performed in terms of both training and model evaluation. Further discussion regarding each stage in the stated process flow is presented in the following sections.

### Data gathering

Images were obtained from different sources and under various conditions in terms of image quality and PPA state. As shown in Table 1, five publicly available databases were collected along with a local dataset, and each is explained in further detail in the following subsections.

**Table 1:** Details of the obtained datasets.

Name	Year of Availability	Number of Images	Format	Location
**RIGA (Bin Rushed)** [25]	2018	195	jpg	Saudi Arabia
**RIGA (Magrabi)** [25]	2018	95	tif	Saudi Arabia
**HRF** [26]	2013	45	jpg	Germany, Czech
**Kaggle** [27]	2018	1,000	jpg	China
**ORIGA (-light)** [28]	2010	650	jpg	Singapore
**Eyepacs** [29]	2015	Around 80,000	jpg	X
**KAIMRC** [30]	2022	2,084	jpg	Saudi Arabia

#### Retinal fundus Images for Glaucoma Analysis (RIGA)

The dataset provides annotations for the optic cup and disc boundaries by six different ophthalmologists. This dataset consists of three files from three different sources: MESSIDOR, Bin Rushed, and Magrabi. Files from this dataset consisted of 460, 195, and 95 fundus images from MESSIDOR, Bin Rushed, and Magrabi, respectively. However, only the Bin Rushed and Magrabi files were used for the study.

#### High Resolution Fundus (HRF) database

The obtained dataset contains 45 images: 15 each of healthy persons, diabetic retinopathy patients, and glaucoma patients. The dataset was provided by the Pattern Recognition Lab (CS5), Department of Ophthalmology, Friedrich-Alexander University Erlangen-Nuremberg (Germany), and Brno University of Technology, Faculty of Electrical Engineering and Communication, Department of Biomedical Engineering, Brno (Czech Republic).

#### Publicly available fundus images dataset—Kaggle repository

The fundus images obtained from the Kaggle repository [27] were made visible to the public. It consists of 1,000 fundus images categorized into 39 classes. This dataset is a subset of a larger database of 209,494 images, and the copyrights for these images belong to the Joint Shantou International Eye Centre (JSIEC), Shantou City, Guangdong Province, China.

#### ORIGA (-light)

The ORIGA (an online retina fundus image database for glaucoma analysis and research) [28] is a dataset of 650 retinal images, which are annotated by professionals from the Singapore Eye Research Institute. These images were collected in a population-based study, the Singapore Malay Eye Study (SiMES).

#### Eyepacs

A publicly available database of fundus images, obtained from various sources and under different acquisition settings and imaging conditions, was provided in the Kaggle repository [29]. This dataset was created to characterize the competition for developing an automated grading system for diabetic retinopathy. Images were separated in the form of training and testing.

#### King Abdullah International Medical Research Center (KAIMRC)

Locally gathered fundus images were obtained from the King Abdullah International Medical Research Center (KAIMRC) [30] in the Ministry of National Guard’s local hospital in Riyadh, Saudi Arabia. This dataset consists of 2,084 fundus images collected from the glaucoma clinic and the retina clinic were 1,600 and 484, respectively.

### Pre-processing

Image labeling and region of interest (ROI) extraction were the two main pre-processing preparation operations performed.

#### Labeling

Because there is no publicly available fundus image dataset labeled in terms of the presence or absence of PPA in the fundus images, the images were categorized visually by certified ophthalmologists and optometrists into two classes: PPA and non-PPA images, without manual annotation of the PPA boundaries. The inter observer variability was considered between an ophthalmologist and senior optometrist. The first labelling round was conducted by the ophthalmologist and then the second round was blindly conducted by the optometrist using Microsoft surface pro with screen size of 12 inches. However, not all the images obtained from public sources were labeled because of the quality of some images (bad acquisition, pathological conditions such as cataract). Additionally, some images were not used because of the mislocalization of the ROI. Table 2 presents the number of images for each class in the datasets.

**Table 2:** A summary of the labeled images.

Source	Total Number of Images	Utilized Number of Images	Non-PPA Images	PPA Images
**RIGA (Bin Rushed)** [25]	195	195	57	138
**RIGA (Magrabi)** [25]	95	94	21	73
**HRF** [26]	45	45	9	36
**Kaggle** [27]	1,000	495	244	251
**ORIGA (-light)** [28]	650	371	49	322
**Eyepacs** [29]	Around 80,000	487	487	x
**KAIMRC** [30]	2,084	2,084	1,178	906

#### Region of Interest (ROI) extraction

The images were cropped in the region of the ONH and considered as the ROI to eliminate the effect of any existing artifacts and unnecessary details for PPA classification and to lower the cost of the computations. The selected architecture is the mask R-CNN [31] modality, which is the new generation of the Faster R-CNN [32] network i.e. an extra head was added to provide instance segmentation capability. The Mask R-CNN is widely used as an off the shelf object detection algorithm for a variety of computer vision applications and it performs well with usually minimal modifications. To perform the modifications for the ROI extraction, the number of training epochs was empirically set as 15 epochs for considering training the model for a decent amount of epochs as well as not too many epochs to avoid the problem of overfitting. Additionally, the conducted algorithm was selected after pretraining it on a large object detection dataset known as Microsoft Common Objects in Context (COCO) dataset. Therefore, we retrained the downstream task (heads) of the model to be fine tuned during the pre training to denote the research objective which considers the detection of the Region of Interest (ROI) in the fundus image. There was no need for more tuning to the model parameters and modifications for further improvement since it performs well for detecting enough of the denoted regions. As a result, the denoted regions were prepared for the main objective of the study, which is classifying the ROI into being positive with the PPA or not.

The architecture of the mask R-CNN consists of a backbone for feature extraction, followed by a region proposal network (RPN) and three head branches; the first one was for classification (providing object classes), the second was for detection (generating bounding boxes), and the last one was for instance segmentation (creating masks for the object of interest). However, only the detection head was used to perform the localization operation for the ROI.

All images are from different sources, thus, setting the appropriate bounding box size to localize the ROI was optimized based on the visibility of the region around the ONH, i.e. to cover an entire suspected PPA area. The ONH structure varies from case to case. Moreover, the fundus images with the same resolution have a few differences because of the visual observation of the cropped images. Hence, the candidate area of the PPA could not be observed. Therefore, an assessment process was performed to ensure that the required region was cropped appropriately.

**Fig 2:**
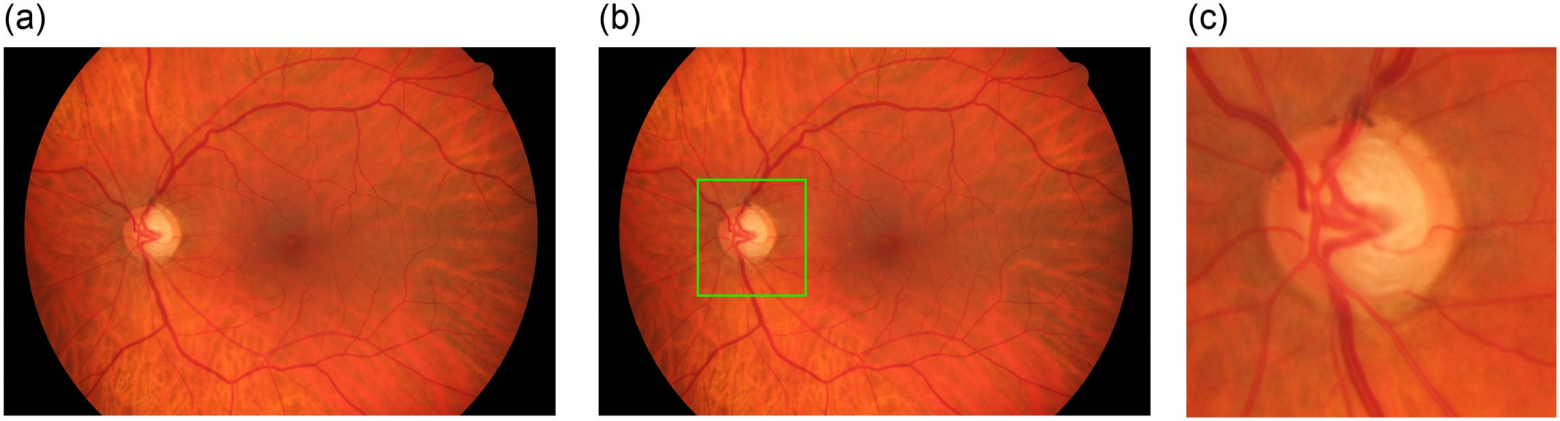
An example of cropping a candidate ROI from a fundus images following the proposed localization approach. In (a) a fundus image is shown, while (b) and (c) present the localized image by the deep learning algorithm and the cropped ROI, respectively.

**Fig 3:**
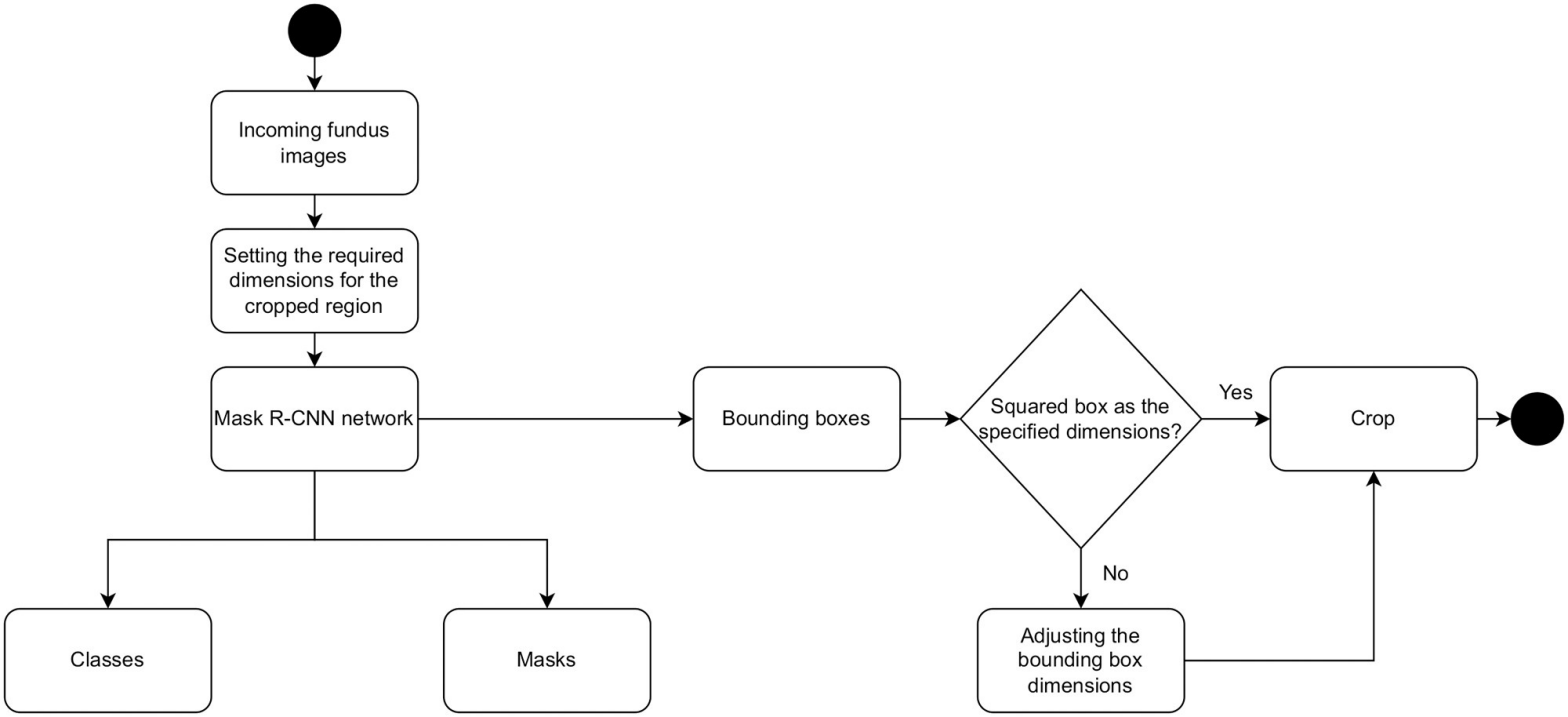
A proposed algorithm to maintain the aspect ratio for all generated bounding boxes.

The deep learning approach provides the predicted coordinates for the ROI. The resolutions of the images and their aspect ratios were considered while setting the dimensions of the cropped regions. However, to maintain the aspect ratio for the cropped images while rescaling these images in further development stages, an algorithm was developed. The bounding boxes generated from the object detection network do not have the same resolution. Therefore, the boxes’ dimensions modified to be squared boxes make them all have the same aspect ratio equal to one. The process was conducted by stating the required cropped region width and height in advance prior to the inference of the region coordinates. Therefore, when obtaining the resultant bounding box, its size is modified to match the specified dimensions by expanding either the x or y dimensions, or both, to denote that specification. Fig 2 presents an example of cropping the detected ROI, while Fig 3 presents the proposed algorithm in accordance with that objective.

All data sets were categorized based on the difference in image resolutions to ease the process of localizing the ROI and setting the appropriate cropping dimensions. Table 3 presents an analysis of the localization model results as follows: localized, multiple localized, and non-localized. The localized feature refers to the number of images for each dataset where the ROI was localized, while the multiple localized feature indicates the number of images where more than one region was localized aside from the localization of the optic nerve head owing to artifacts in fundus images and fringe presence. However, from multiple localized regions, a manual assessment was performed to eliminate images rather than the ROI (167 images [0.6%]). The non-localized feature represents the number of images where no region was detected owing to the unclear conducted image of the ROI. The localization model provides a localization rate of 98.103% and 95.681% for images obtained from public sources and the KAIMRC dataset, respectively, which resulted in only 32 missed images out of the used 1,687 public images and 90 images from KAIMRC.

**Table 3:** A summary of the analysis for the ROI cropping stage.

Source	Total Images	Used	Localized	Multiple Localized	Not Localized	Localization Rate
**RIGA–BinRushed** [25]	195	195	183	9	3	98.461%
**RIGA–Magrabi** [25]	95	94	86	8	0	100.00%
**HRF** [26]	45	45	39	1	5	88.88%
**Eyepacs** [29]	487	487	451	25	11	97.741%
**ORIGA** [28]	650	371	350	16	5	98.652%
**Kaggle** [27]	1000	495	379	108	8	98.383%
**Total**	2472	1687	1488	167	32	98.103%
**KAIMRC–Glaucoma**	1600	1600	1388	150	62	96.125%
**KAIMRC–Retina**	484	484	410	46	28	94.214%
**Total**	2084	2084	1798	196	90	95.681%

### Convolutional neural network classification model

The CNN is the selected deep learning algorithm for the proposed classification system [33]. CNNs are capable of performing well with challenging computer vision tasks because they consist of several complex layers that provide these models with the ability to extract significant features that distinguish different objects [34]. As part of the deep learning algorithm nature, the top layers of any network can detect general features, such as edges, corners, and curves, while the deep layers are capable of learning about the specific features of the dataset in use. However, these types of algorithms require several images to operate perfectly. Because a lack of data and the cost of labeling large datasets are common limitations in the design of machine learning algorithms, the transfer learning technique has been used.

**Fig 4:**
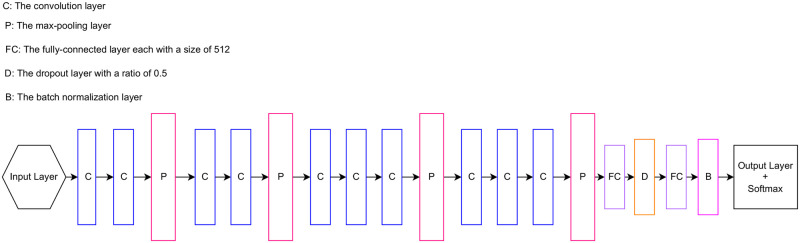
The proposed PPA classification model architecture.

In this study, we used the backbone of the VGG16 [35] network with pre-trained weights using the ImageNet dataset. Referring to the VGG16 [35] network architecture, its backbone consists of five blocks. Several layers exist within these blocks, such as convolution, max pooling, and activation functions. The proposed model architecture consists of the first four blocks of the backbone, while the first three blocks were kept frozen with the pre-trained weights of the ImageNet dataset during the training process to use those weights to extract the general features from the training images in our dataset, while the last block of the backbone was re-trained on the used dataset for the conducted problem. Additionally, two fully connected layers were added after the backbone, and each classification layer was followed by a ReLU activation function. Dropout and batch normalization layers were added, acting as regularization layers. The final classification layer was changed completely, as per the problem requirement, into a binary classification layer, followed by a Softmax activation function. The characteristics of the proposed network architecture are shown in Fig 4.

## Evaluation protocol

### Performance measures

The classification model was evaluated using five performance measures: precision, recall, F1-score, accuracy, and balanced accuracy.

**Fig 5:**
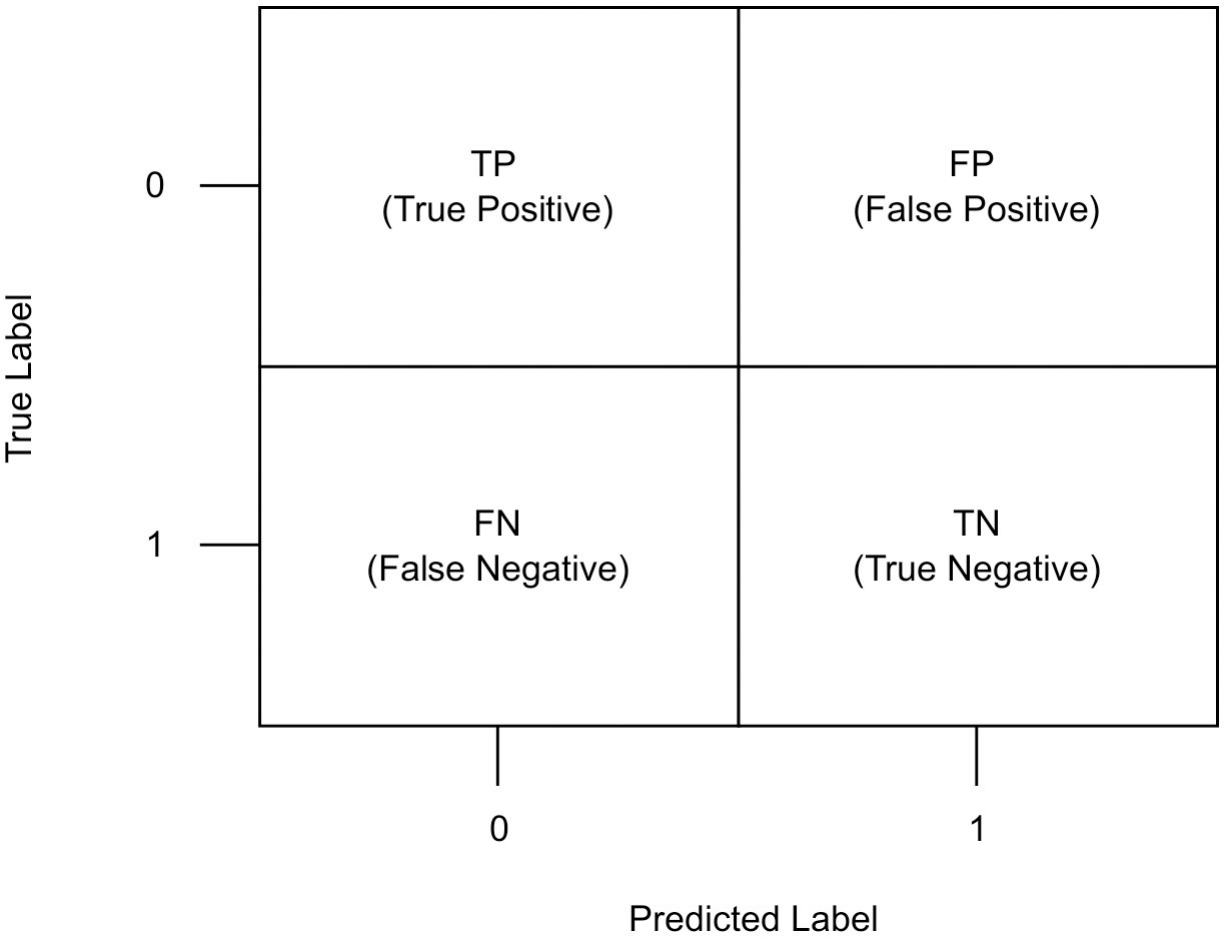
Confusion matrix diagram.

The confusion matrix is an evaluation mapping criterion for extracting the required parameters to calculate the measured values. The interpreted parameters for calculating these metrics are true positive, true negative, false positive, and false negative, as shown in Fig 5. The accuracy, precision, recall, and F1-score formulas are presented in Eqs 1–4. Additionally, because the accuracy measure is sensitive to the class imbalance issue, an addendum evaluation, balanced accuracy, was used. and its formula is shown in Eq 5. Additionally, the K-fold cross-validation approach was used to evaluate the proposed model to assess the reliability of the results.


$$Accuracy=\;\frac{TP+TN}{TP+TN+FP+FN},$$ (1)

$$Precision=\;\frac{TP}{TP+FP}$$ (2)

$$Recall=\;\frac{TP}{TP+FN}$$ (3)

$$F1-Score=2\;\times \left(\frac{Precision\;\times Rcall}{Precision\;+\;Recall}\right)$$ (4)

$$Balanced\;Accuracy=\;\frac{1}{2}\;\times \left(\frac{TP}{TP+FN}+\;\frac{TN}{TN+FP}\right)$$ (5)

### Implementation details

#### Selected parameters

The implementation of the mask R-CNN by Matterport [36] was the selected approach for the process of localizing the ROI within fundus images. No major adjustments were made for the network hyperparameters, except for the number of epochs (15 epochs). The top layers of the model were re-trained for the KAIMRC dataset images by randomly selecting 200 images for both training and testing purposes. The required ground truth for the model to be trained and evaluated with was generated manually using Labelimg software by annotating the ROI. The process of creating the ground truth was cautiously performed by observing all images and ensuring that an appropriate region from all sides was annotated. Additionally, 200 images were used for training the model with 160 images, and the remaining 40 images were used to evaluate the model. However, because the model performance for the prediction states was good, as shown in Table 3, there is no need to further tune the model and keep improving the results. The model was implemented in the Google Collaboratory Cloud platform with the required versions of libraries and platforms, as stated in Matterpor [36].

To select the best architecture for the classification model, three sets (public sources (better resolution quality), KAIMRC images (lower resolution quality), and a combined set (public sources and KAIMRC images) were used to obtain comparison results to analyze the model’s performance.

Several experiments were conducted to obtain the best model. The architectures of various models were experimented with the transfer learning method to overcome the limitation of having small datasets and to use the benefits of having models that are pre-trained with many images. The models used are VGG16 [35], ResNet50 [37], and InceptionV3 [38]. The layers of these models were trained as follows: freezing all layers during the training process and only unfreezing the last few layers.

Additionally, the hyperparameters of the experimented classification models were tuned during development. The learning rate was tuned using three different values: 0.1, 0.0001, and 0.0000001. The batch size was set to 128. Adam optimizer was used as the learning algorithm, and the loss function was used as the categorical cross-entropy. All models were trained for 100 epochs to follow the model selection approach, which involved either selecting the best model across all epochs based on the validation accuracy or the model at the last epoch after all training iterations were completed.

Regarding the splitting methodology, random and stratified fashions were tested. The 80/20 rule was followed, with 80% of the dataset used for training and 20% for testing. Furthermore, the training set was divided into a training and validation set using the same approach as the validation set was used to assess the obtained model before the evaluation stage and to tune the hyperparameters.

While performing experiments and selecting the best classification model, the cross-validation evaluation technique is performed by splitting the used dataset randomly into a pre-identified number of folds. Thus, the model is trained with all folds, except for a reserved fold to be used as the testing fold. This process is repeated by interchanging the folds used to ensure that each fold is conducted for both the training and evaluation of the model, which assesses the reliability of the implemented algorithm.

#### Classification model selection

**Fig 6:**
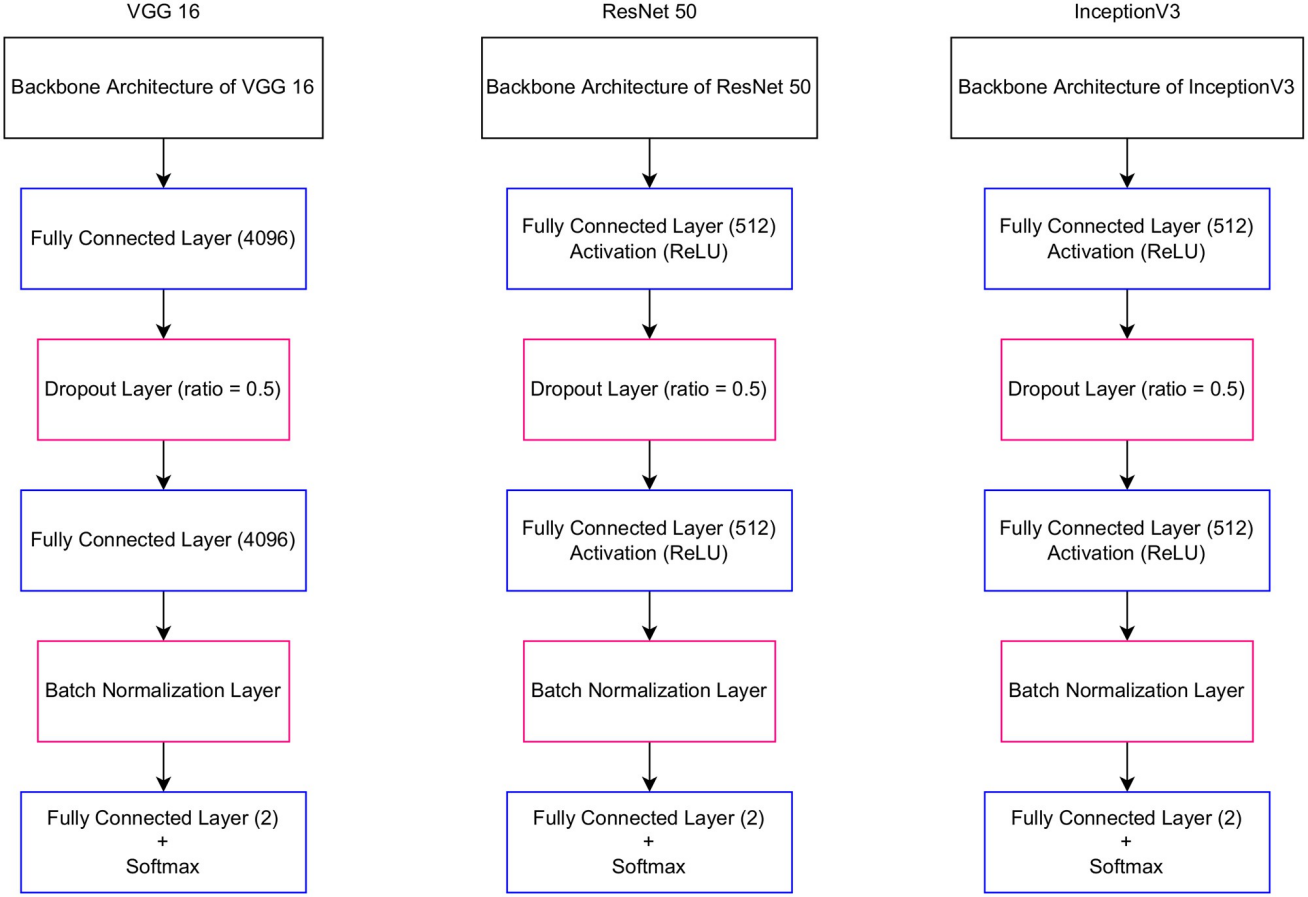
The selected architectures for the performed experiments.

The selection of the best model for the assigned problem was performed after experimenting with three different model architectures. VGG16 [35], ResNet50 [37], and InceptionV3 [38] architectures were selected as experimental networks. Images from public sources and the KAIMRC database were used. Fig 6 presents the baseline architecture for the model selection stage. For the VGG16 [35] model, all layers in the backbone were kept frozen with the ImageNet dataset weights, while re-training the last two fully connected layers. According to He et al. [37], the ResNet50 model consists of only backbone architecture layers and no pre-trained dense layers, similar to the InceptionV3 [38] model. Therefore, two fully connected layers were added for classification with a ReLU activation function following each layer. Additionally, dropout and batch normalization layers were added, along with a fully connected layer with two neurons because of the type of problem, which is a binary classification, followed by a Softmax activation function. In addition to freezing all layers in the backbones of these models, the last few convolution layers were unfrozen.

According to Sharma et al. [23], if the images have different resolutions, all were resized to 224 × 224 for the VGG16 and ResNet50 and to 299 × 299 for the InceptionV3 models after the ROI extraction stage was performed because the used models were pre-trained for the ImageNet weights with the selected spatial dimensions while using the transfer learning technique.

The model was selected after analyzing the results of the model obtained from the last epoch and the model with the best validation accuracy during the training iteration. The analysis encountered evaluation measures. The model results were better when the last convolution layers were unfrozen with all three datasets. The parameters selected for the learning rate and splitting fashion were 0.0001 and random splitting, respectively. The two fully connected layers after the backbone of the network were unfrozen in the process to re-train the weights of these layers for the problem at hand. Additionally, unfreezing the last convolution layers in the network’s backbone, the last block, outperforms the results when all the convolution layers of the model are frozen.

Finally, the model was simplified into a simpler architecture by removing layers from the head of the architecture to reduce the number of trainable parameters in the model. The loss function was changed to hinge loss instead of cross-entropy to mitigate the encountered vanishing gradient problem with the base VGG model.

## Results

All the aforementioned experiments were used to study a machine with the following specifications: 3.60 GHz CPU, 64 GB RAM, and an NVIDIA GeForce RTX 2080 GPU. The software and platforms used for the model implementation are CUDA version 11.0, cudnn SDK 8.0.4, Python 3.7, TensorFlow, and Keras with 2.4.0 and 2.4.3 versions, respectively.

**Fig 7:**
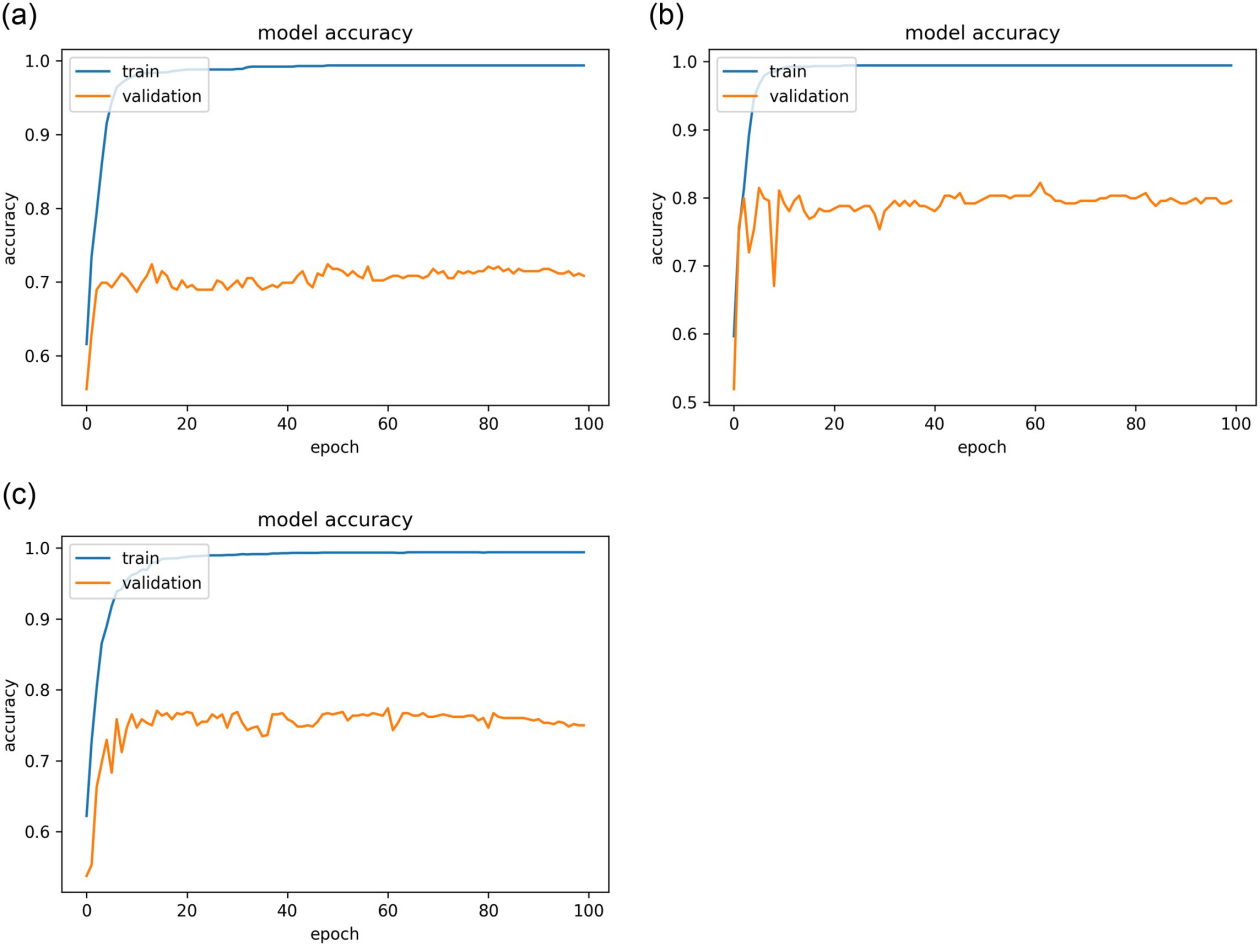
The resultant accuracy curves while using the hinge loss function for the best model. (a) KAIMRC dataset. (b) Public dataset. (c) Combined dataset.

**Fig 8:**
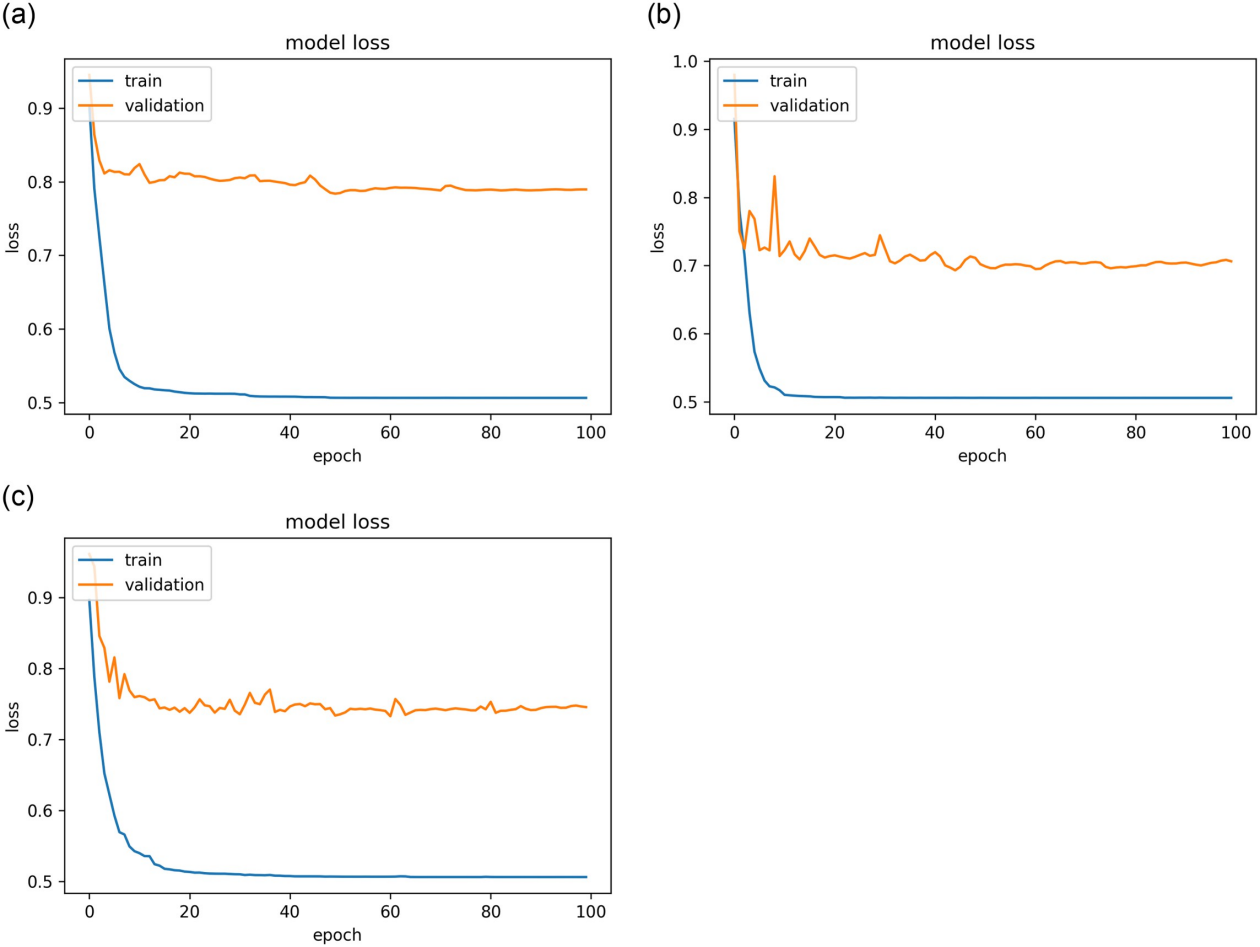
The resultant loss curves while using the hinge loss function for the best model. (a) KAIMRC dataset. (b) Public dataset. (c) Combined dataset.

**Fig 9:**
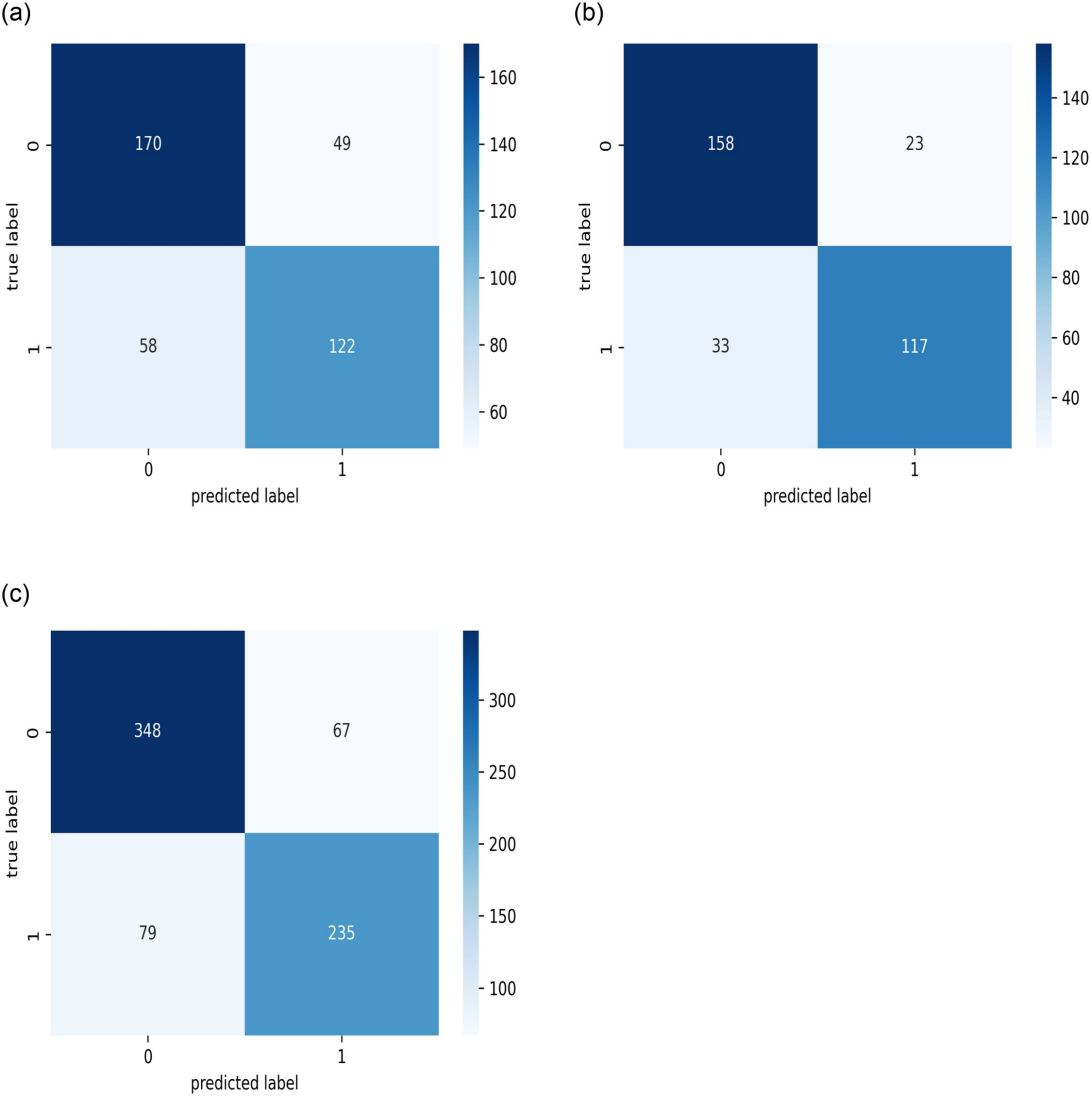
The confusion matrices on the test sets. (a) KAIMRC dataset. (b) Public dataset. (c) Combined dataset.

The results of the best model, as stated for the three datasets (public, KAIMRC, and combined images) are presented in Figs 7–9 and Table 4. The results demonstrate that the VGG16 architecture was adjusted by eliminating the top layers and decreasing the number of trainable parameters, as well as changing the loss function, mitigating the vanishing gradient problem with VGG models.

**Table 4:** The obtained classification reports while evaluating the best model on the testing sets.

KAIMRC Dataset					
**Class Label**	**Precision**	**Recall**	**F1-Score**	**Accuracy**	**Balanced Accuracy**
**Non-PPA**	0.76	0.77	0.77	0.74	0.73
**PPA**	0.72	0.71	0.71		
**Public Datasets**					
**Non-PPA**	0.83	0.87	0.85	0.83	0.82
**PPA**	0.84	0.78	0.81		
**Combined Datasets**					
**Non-PPA**	0.81	0.84	0.83	0.80	0.79

**Fig 10:**
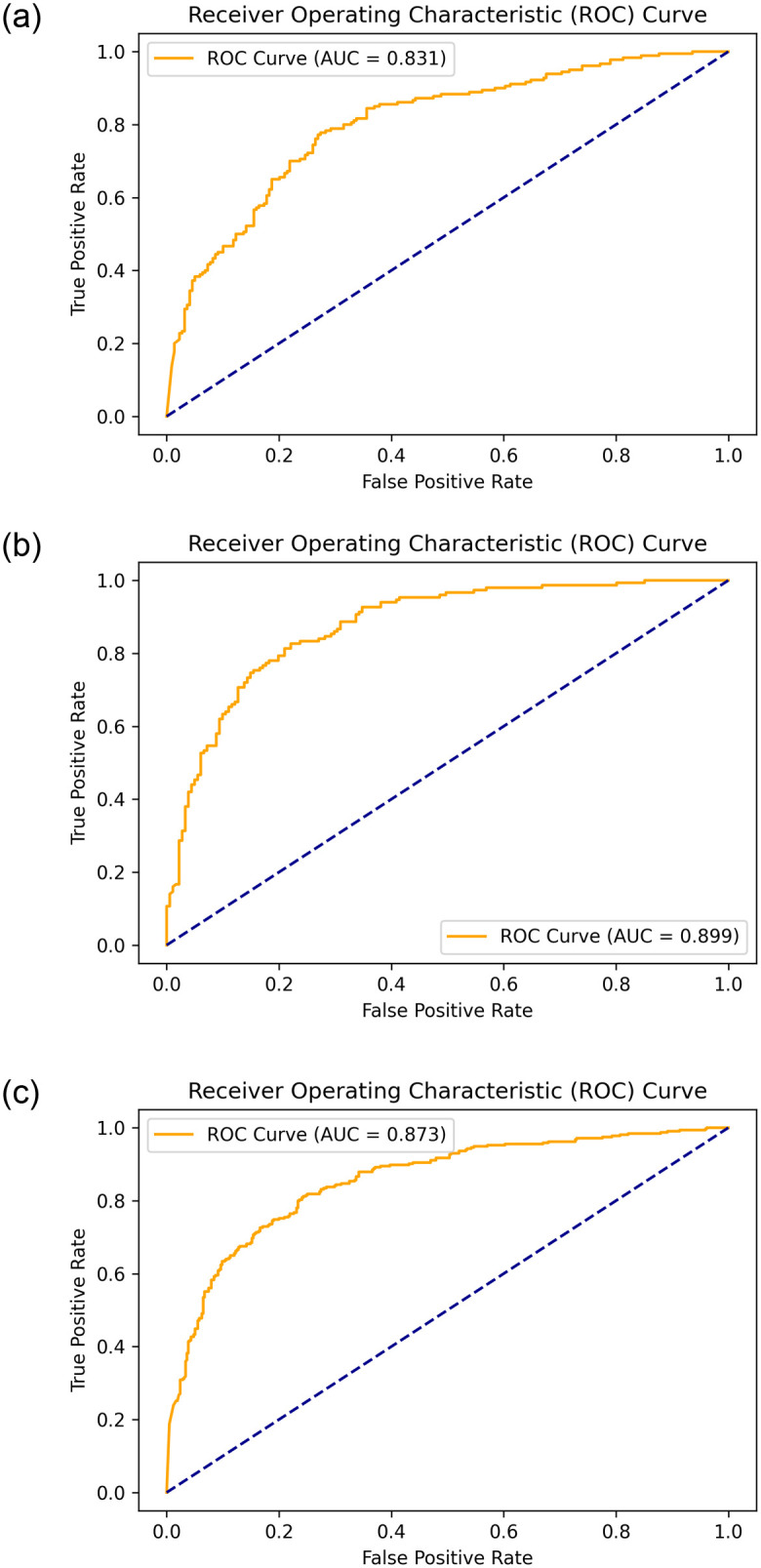
The ROC curve on the three used datasets and showing the resultant AUC score. (a) KAIMRC dataset. (b) Public dataset. (c) Combined dataset.

An addendum evaluation metric was used to evaluate the selected models, which is the ROC_AUC. It is typically used as an evaluation metric for binary classification problems. It also represents the relationship between the true positive rate (TPR) and the false positive rate (FPR) at various threshold values. It was obtained for the best model, as shown in Fig 4. Fig 10 presents the resultant ROC_AUC for the test set for each database. The model yields AUC scores of 0.83, 0.89, and 0.87 for the local, public, and combined datasets, respectively.

**Fig 11:**
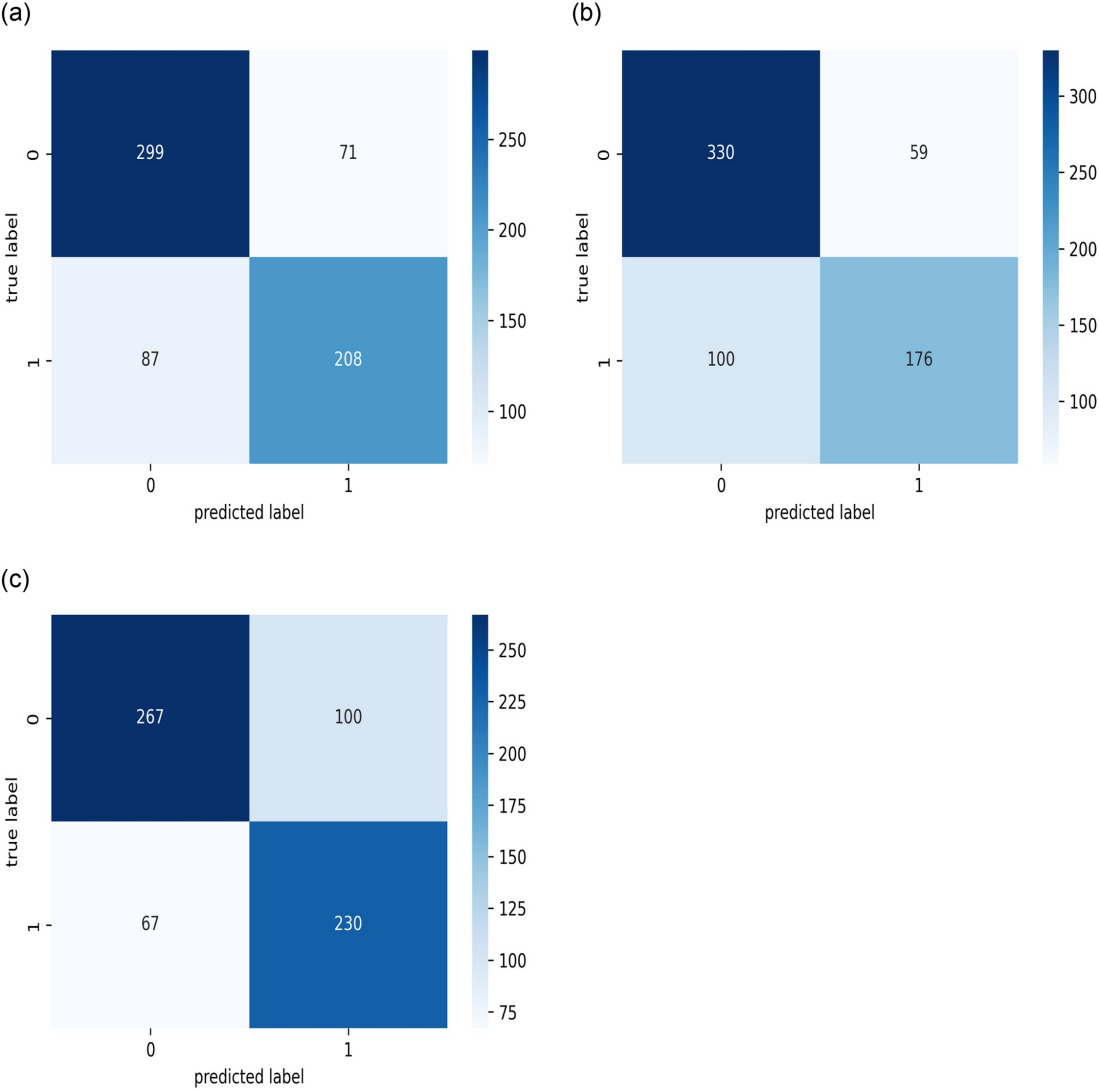
The confusion matrices for the test sets while performing the cross validation on the KAIMRC images. (a) First Fold. (b) Second Fold. (c) Third Fold.

**Fig 12:**
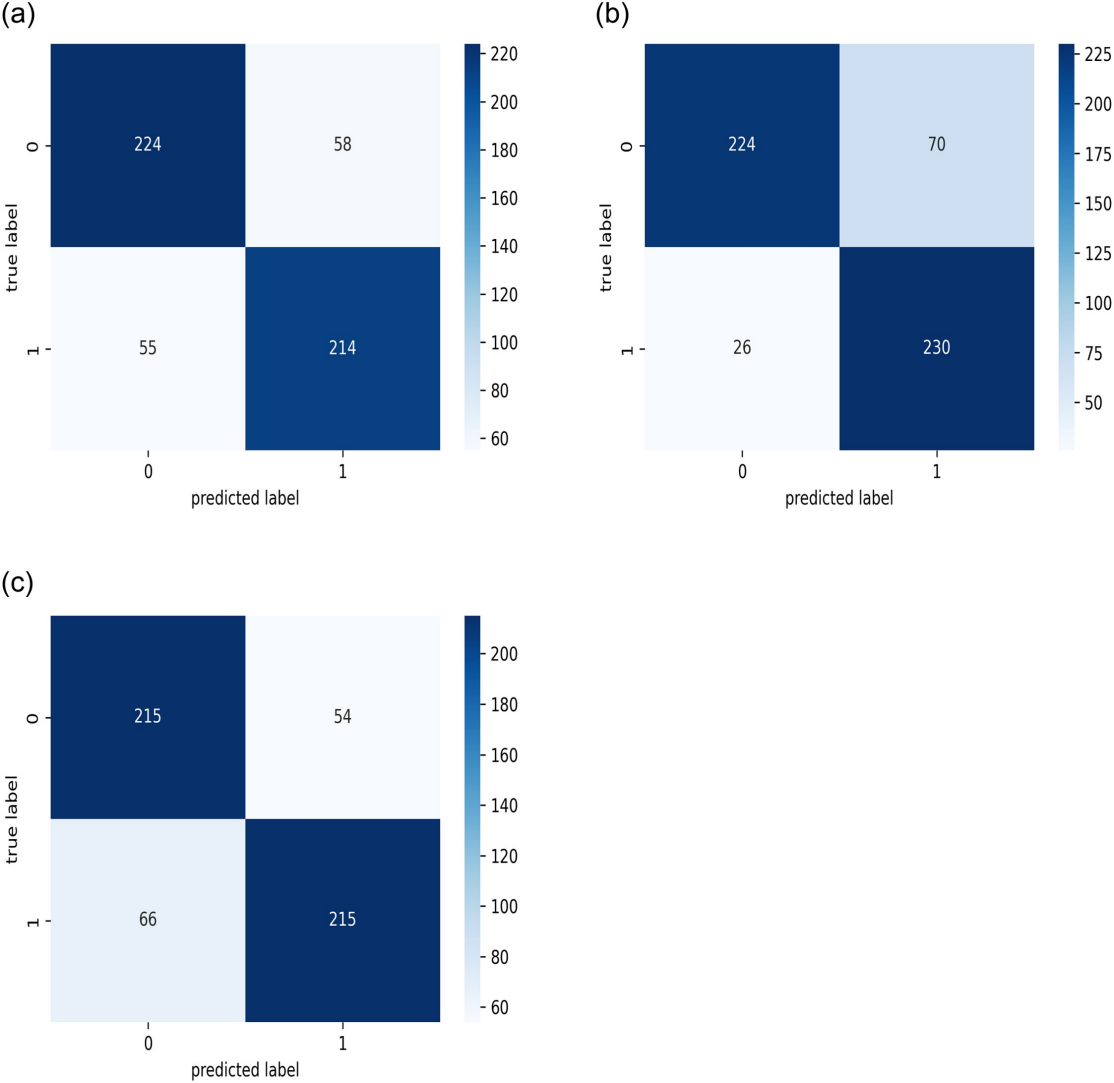
The confusion matrices for the test sets while performing the cross validation on all the obtained public images. (a) First Fold. (b) Second Fold. (c) Third Fold.

**Fig 13:**
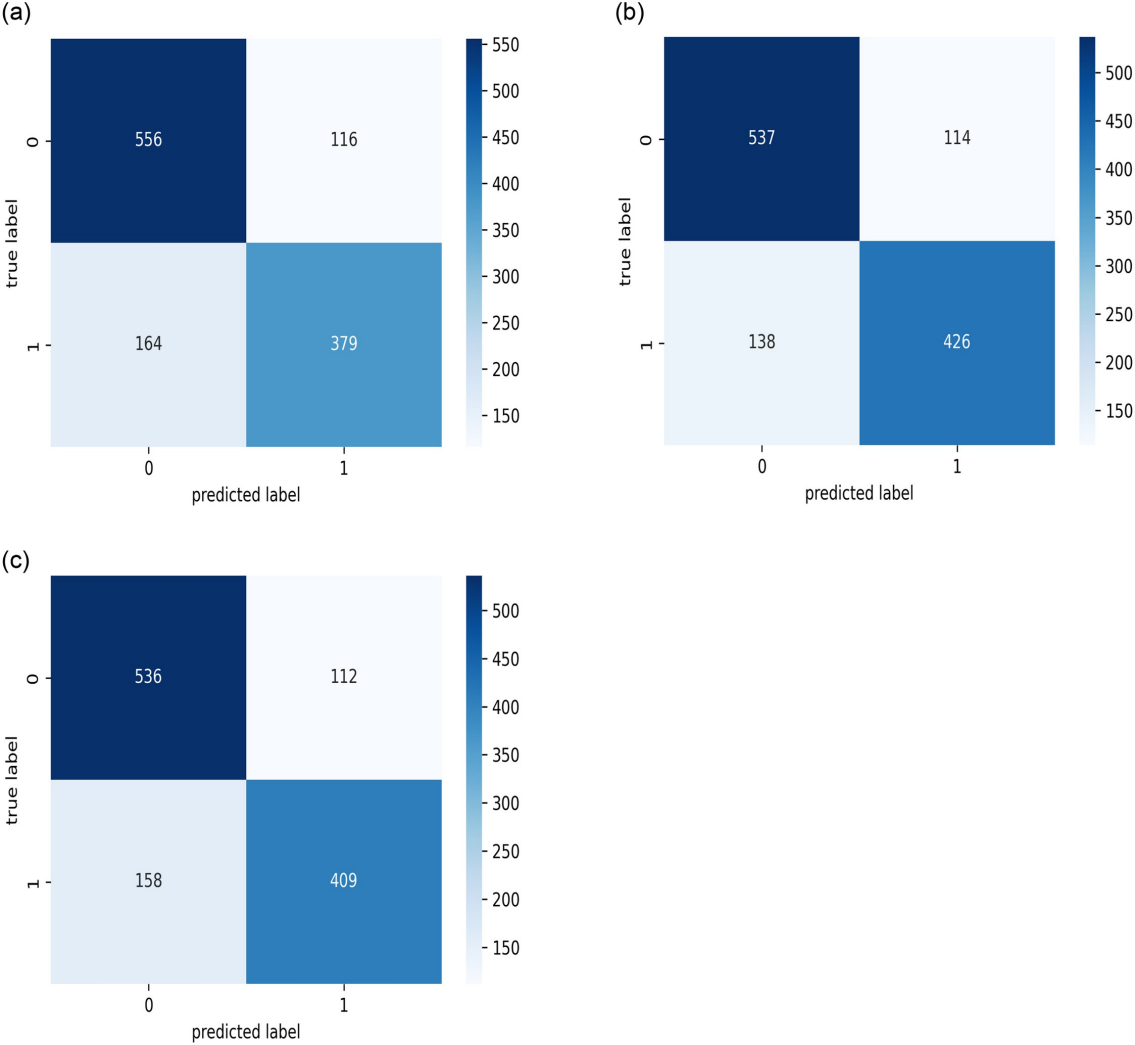
The confusion matrices for the test sets while performing the cross validation on the combined images from both public sources and KAIMRC database. (a) First Fold. (b) Second Fold. (c) Third Fold.

**Fig 14:**
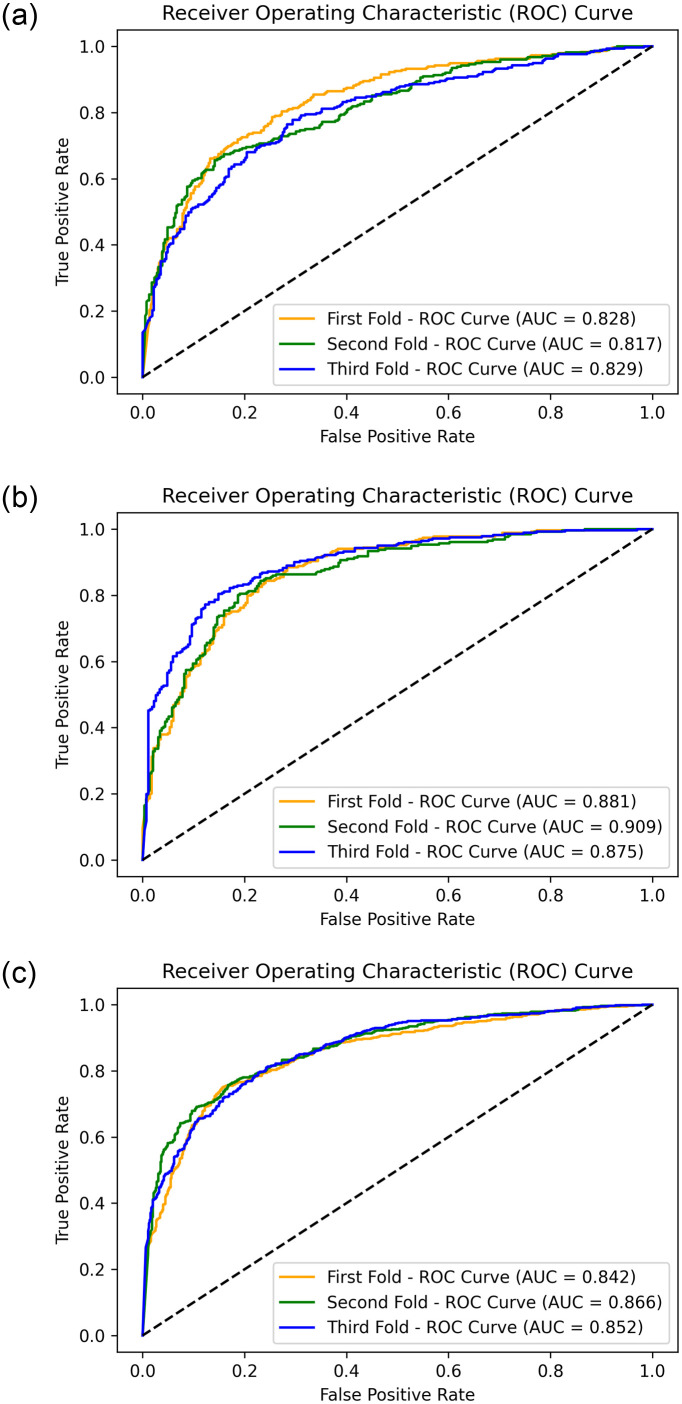
The ROC curves while performing the cross validation on the three datasets. (a) KAIMRC images. (b) Obtained Public images. (c) Combined images.

Additionally, because the development process used only one-fold of the datasets, K-fold cross-validation was used, with a value of three selected for the number of folds. Figs 11–13 present the resulting confusion matrices while training and testing the best model with each dataset for the three folds. Fig 14 shows the ROC_AUC curves for the three folds while conducting the KAIMRC, public, and combined sets, respectively, and Tables 5–7 show the classification reports obtained.

**Table 5:** The obtained classification reports for the test sets after performing the cross-validation on the KAIMRC images.

Fold No. 1				
	**Precision**	**Recall**	**F1-score**	**Balanced Accuracy**
**Non PPA**	0.77	0.81	0.79	0.75
**PPA**	0.75	0.71	0.72	
**Fold No. 2**				
	**Precision**	**Recall**	**F1-score**	**Balanced Accuracy**
**Non PPA**	0.77	0.85	0.81	0.74
**PPA**	0.75	0.64	0.69	
**Fold No. 3**				
	**Precision**	**Recall**	**F1-score**	**Balanced Accuracy**
**Non PPA**	0.80	0.73	0.76	0.75
**PPA**	0.70	0.77	0.73	
Average Accuracy = 0.75 (+ - 0.62)				

**Table 6:** The obtained classification reports for the test sets after performing the cross-validation on the public images.

Fold No. 1				
	**Precision**	**Recall**	**F1-score**	**Balanced Accuracy**
Non PPA	0.80	0.79	0.80	0.79
PPA	0.79	0.80	0.79	
Fold No. 2				
	**Precision**	**Recall**	**F1-score**	**Balanced Accuracy**
Non PPA	0.90	0.76	0.82	0.83
PPA	0.77	0.90	0.83	
Fold No. 3				
	**Precision**	**Recall**	**F1-score**	**Balanced Accuracy**
Non PPA	0.77	0.80	0.78	0.78
PPA	0.80	0.77	0.78	
Average Accuracy = 0.80 (+ - 1.82)				

**Table 7:** The obtained classification reports for the test sets after performing the cross-validation on the combined images.

Fold No. 1				
	**Precision**	**Recall**	**F1-score**	**Balanced Accuracy**
**Non PPA**	0.77	0.83	0.80	0.76
**PPA**	0.77	0.70	0.73	
**Fold No. 2**				
	**Precision**	**Recall**	**F1-score**	**Balanced Accuracy**
**Non PPA**	0.80	0.82	0.81	0.79
**PPA**	0.79	0.76	0.77	
**Fold No. 3**				
	**Precision**	**Recall**	**F1-score**	**Balanced Accuracy**
**Non PPA**	0.77	0.83	0.80	0.77
**PPA**	0.79	0.72	0.75	
Average Accuracy = 0.78 (+ - 0.95)				

## Discussion

Because of the similarity in intensity, distinguishing PPA and OD boundaries using fundus images is difficult. However, the accomplished work for the PPA symptom generally involves hand-crafted methods, which lack the general feature because different datasets were used while implementing each algorithm, potentially introducing a performance degradation when using the same algorithm with different datasets. Nonetheless, incorporating data from multiple sources with varying PPA stages is vital for developing a robust detection algorithm that has better generalizability. Therefore, we have provided a methodology for PPA detection in fundus images, which uses complete deep learning approaches without any image processing or hand-crafted methods using various datasets from both public and local sources.

### Performance of deep learning

Current state-of-the-art PPA detection models combine hand-crafted and deep features or suffer from small PPA data that hinder their generalizability [21–24]. Cheng et al. [21] proposed an algorithm by extracting biologically inspired features from images. A fringe removal-based approach was applied to localize the OD. A total of 1,584 images from the Singapore Cohort study of the risk factors for myopia (SCORM) were used to train and test the model. The model achieved 90% accuracy in detecting the PPA. In this study, the lack of PPA in glaucoma or adults indicates a lack of diversity in the images. Other signs may be present in the glaucomatous cup, and the use of this model in glaucoma patients may not be as accurate. However, such an approach might result in performance degradation while performing the same localization and detection methods as other images from different datasets. Septiarini et al. [22] proposed a methodology for the PPA classification problem over three different datasets using a thresholding approach for ROI localization and a backpropagation neural network for image classification. These datasets were obtained from different sources; however, the number of images was limited because only 155 fundus images were used. The lack of various images in terms of quality and clinical conditions would introduce a limitation while performing the same methodology with other datasets for either ROI localization or classification part of the system. The accuracy was reported for the stated algorithm as 96% overall, while 100% accuracy was achieved with severe cases. Sharma et al. [23] used six public datasets and labeled them into three categories: healthy, PPA, and others. Only the first two labels were used in their study. A hand-crafted method was used in parallel with a deep learning model, ResNet50, which introduced some limitations in the model. The hand-crafted method employs image features extracted from the gray level co-occurrence matrix (GLCM), as well as their homogeneity, correlation, and image contrast. Therefore, even though various images were obtained, a hand-crafted method was still used to localize the ROI and in the classification part of the algorithm. The performance was reported with average sensitivity, specificity, and accuracy values of 95.83%. An addendum study was revised to introduce texture analysis for the PPA identification objective [24]. A thresholding approach was used to localize the region where the PPA occurred; however, limited data of only 80 retinal fundus images were available during the experiments. The sensitivity for detecting moderate to severe PPA regions was 73%, with a specificity of 95%.

Our work presents a two-stage automated end-to-end deep learning system for ROI localization and PPA classification. Sharma et al. used various datasets with comparable diversity in their study [23]. Hence, this work overcomes the limitations in the literature, namely, the use of hand-crafted methods and small datasets. A deep learning model of the mask R-CNN architecture was re-trained for the problem assigned and for the classification part of the method, because no features were algorithmically extracted from the images. Additionally, a deep learning model used for both feature extraction and classification stages.

Several performance measures were conducted for the PPA classification stage, which increased the reliability of the reported performance. Six metrics were obtained for the model. In addition to these performance measures, the K-fold cross-validation evaluation approach was performed, and no study has been conducted using this approach. Furthermore, as shown in Table 4, the results for the public datasets were better than either the KAIMRC images or the combination of all images. This is because public images have better quality than the KAIMRC. Even when the number of used public images is less than the number of images in the KAIMRC database, it outperforms its results by a significant margin. When combining all images into a single dataset called the combined dataset, the results remain lower than the model with the public images, which is caused by the effect of including the KAIMRC images.

The main contribution of this study is the development of a system by designing the proposed approach through various datasets to provide a more robust and generalized model, particularly when performing the K-fold cross-validation evaluation methodology because the variation of the model results across all images in the conducted dataset is reported. Obtaining such a system to expedite the detection of the progression of glaucoma and other related diseases will aid clinicians, particularly in primary clinics or regions where there are limited clinical experts.

### Limitations and future work

This study had some limitations. It used a complex deep learning system to classify PPA versus non-PPA; therefore, an interpretable surrogate model can be used to explain how it reached its prediction. In future efforts, one can experiment with the operating point (classification threshold) in the training set to find the optimal threshold that maximizes the Youden index with ROC curve analysis. Moreover, using more data for the problem at hand can be addressed by either obtaining more diverse images with PPA states or mitigating the cost of labeling limitation. Further improvement of the developed algorithms while performing either localization for the ROI or the classification model is needed. The number of missed images can also be mitigated to achieve better results for the identification of PPA features using the classification model.

## Conclusion

Early detection of PPA improves the diagnosis of glaucoma using a combination of other clinical indicators. In this study, a PPA detection approach was developed with two deep learning models operating in sequence to localize the region where the PPA occurs as a preprocessing stage, thus classifying that area while conducting various dataset images. The implementation of the localization model was obtained using the Matterport [32] implementation for the mask R-CNN network. A classification architecture is proposed for classifying localized regions based on whether a PPA is developed. For the localization stage, the results were reported while evaluating the experiments performed with images from different distributions. For the classification network, the transfer learning approach with pre-trained weights on the ImageNet dataset was used. In this study, five public sources were used in addition to the images gathered from a local source, which resulted in an overall diverse database.

Future work is proposed for the developed system in terms of both localization and classification methodologies. The localization model results include some duplicated detected regions, which can be resolved by either tuning the used network or developing an image processing approach. Several performance measures have been reported for the classification model. The results can be improved using different deep learning architectures and more images.
